# Supplementary material for: Balancing typological and dimensional approaches: Assessment of adult attachment styles with Factor Mixture Analysis
Source: PLoS One. 2021 Jul 8;16(7):e0254342. doi: 10.1371/journal.pone.0254342 (PMC8266088; doi:10.1371/journal.pone.0254342)
Supplement: Attachment — Submitted filename: Response to Reviewers.docx [file pone.0254342.s001.docx]

RESPONSE TO REVIEWERS

Editor:

1. As the editor suggests, we have reviewed the PLOS ONE's style requirements, and we have carried out several corrections. Furthermore, we have changed the names of the files for Figure 1 and Figure 2.
2. We have reviewed our reference list to ensure that is correct and complete. In fact, we have made several changes in the references to meet the PLOS ONE's style requirements. Furthermore, we have added the new reference suggested by Reviewer 1 (doi: 10.1002/imhj.20094). We have changed one of our previous references (doi: 10.5944/ap.9.1.435) for another one (doi: 10.1016/j.rips.2016.05.002), because the doi of the previous reference does not link properly with the article. On the other hand, we have not cited any paper that have been retracted.
3. All the participants were undergraduate students older than 17. In fact, this study is focused on **adult** attachment styles, and for this reason one exclusion criteria to participate in this study was being under 18 years old, as we specified to the research ethics committee. We have corrected the mistake we made in the Participants’ section for which we apologize.
4. We have uploaded our data in the ZENODO repository. The doi is https://doi.org/10.5281/zenodo.4923526
5. We have removed the section “Supporting information” on page 34, because we have not supporting tables or figures.

Reviewer 1:

We are grateful to the reviewer for his/her useful recommendations. We have made several changes in the manuscript, following these comments and suggestions, as we explain below:

- As the reviewer suggests, we have described and referred the instrument *Relationship Questionnaire – clinical version RQ–CV* in the Introduction section. More specifically, we have explained that it includes an additional attachment-style description, labeled profoundly-distrustful attachment style. We also explain than it is a simple instrument, easy to use, and which also encompasses both categorical and dimensional perspectives. Indeed it is of potential interest for further studies.
- We also agree with the reviewer in that it is important to use both the categorical and the dimensional perspectives if a deeper understanding about adult attachment styles is to be attained. In fact, although some adults may fit primarily into one category, as the reviewer says, at the same time they may exhibit some characteristics on some other category. For this reason, the dimensional perspective complements the categorical one. We have explained this point in the revised version of the manuscript.
- The current study is not focused on gender differences in validity relations, but it would be interesting to carry out further studies to determine whether the gender differences found by Wilhelm et al. are replicated with the instruments and the methodology of the current study. We have explained this point in the manuscript.
- We agree with the reviewer in that having only students in our sample is a limitation, as we recognized in the manuscript. For this reason, we consider that further studies are needed with more heterogenous samples, in order to determine if more profiles can be identified, among them the dismissive/independent attachment style. However, we believe that most adults will probably belong to the undifferentiated profile, as found in the present study, and that only few of them will be in the dismissive/independent or anxious/avoidant styles.
- We have reviewed the manuscript in order to reduce some repetitions, and we have cut down some words in the lines indicated by the reviewer. We are grateful for this suggestion and the other remarks.

Reviewer 2:

We are grateful for the positive and encouraging comments of the reviewer. As he/she says, FMA is a complex modeling and this involves some limitations that are unavoidable if identifiable and stable solutions are to be obtained. However, it should be taken into account that the two-class solution has clearly a better fit than the other competing solutions, and the results of both the FMA and validity analyses are consistent, as the reviewer recognizes. Therefore, we consider that the two-class solution was an appropriate choice.

The reviewer considers that, after so many previous multiple-classes and taxometric proposals, the simple FMA results we obtained here are somewhat disappointing. We understand this view and partly agree with it, but we would like to stress that our conclusions are plausible and well supported by the results found in this study, as the reviewer acknowledges. It is quite possible that more profiles could be obtained with a more heterogeneous sample, or with other instruments with a clearer factor structure. But, at the same time, we believe that this “finer” distinctions would be relatively minor and would not contradict the present results. We would like to stress again that this appears to be the first study in the field that is based on FMA, and that, compared to previous approaches, this analytical tool is considered to be more adequate for encompassing both the categorical and dimensional approaches. Overall, we consider the current study as a relevant starting point that provides solid bases results. However, further studies based on more heterogeneous samples and other instruments most widely used in this field are clearly needed in order to determine whether a larger number of profiles can be identified.
